# Recent advances on anti-angiogenic multi-receptor tyrosine kinase inhibitors in osteosarcoma and Ewing sarcoma

**DOI:** 10.3389/fonc.2023.1013359

**Published:** 2023-03-13

**Authors:** Emmy D. G. Fleuren, Myrella Vlenterie, Winette T. A. van der Graaf

**Affiliations:** ^1^ Children’s Cancer Institute, Lowy Cancer Research Centre, UNSW Sydney, Sydney, NSW, Australia; ^2^ School of Clinical Medicine, UNSW Medicine and Health, UNSW Sydney, Sydney, NSW, Australia; ^3^ Centre for Childhood Cancer Research, UNSW Sydney, Sydney, NSW, Australia; ^4^ Department of Medical Oncology, Netherlands Cancer Institute, Amsterdam, Netherlands; ^5^ Department of Medical Oncology, Erasmus MC Cancer Institute, Erasmus University Medical Center, Rotterdam, Netherlands

**Keywords:** osteosarcoma, Ewing sarcoma, tyrosine kinase, tyrosine kinase inhibitor, angiogenesis

## Abstract

Osteosarcoma (OS) and Ewing sarcoma (ES) are the two most common types of primary bone cancer that predominantly affect the young. Despite aggressive multimodal treatment, survival has not improved significantly over the past four decades. Clinical efficacy has historically been observed for some mono-Receptor Tyrosine Kinase (RTK) inhibitors, albeit in small subsets of OS and ES patients. Clinical efficacy in larger groups of OS or ES patients was reported recently with several newer generation multi-RTK inhibitors. All these inhibitors combine a strong anti-angiogenic (VEGFRs) component with simultaneous inhibition of other key RTKs implicated in OS and ES progression (PDGFR, FGFR, KIT and/or MET). However, despite interesting clinical data, none of these agents have obtained a registration for these indications and are thus difficult to implement in routine OS and ES patient care. It is at present also unclear which of these drugs, with largely overlapping molecular inhibition profiles, would work best for which patient or subtype, and treatment resistance almost uniformly occurs. Here, we provide a critical assessment and systemic comparison on the clinical outcomes to the six most tested drugs in this field in OS and ES to date, including pazopanib, sorafenib, regorafenib, anlotinib, lenvatinib and cabozantinib. We pay special attention to clinical response evaluations in bone sarcomas and provide drug comparisons, including drug-related toxicity, to put these drugs into context for OS and ES patients, and describe how future trials utilizing anti-angiogenic multi-RTK targeted drugs could be designed to ultimately improve response rates and decrease toxicity.

## Background

Osteosarcoma (OS) and Ewing sarcoma (ES) are the two most common types of primary bone cancer that predominantly affect the young. Standard-of-care treatment has not changed much during the past decades, still including a combination of intensive polychemotherapy with surgery, and radiotherapy in ES. Despite this multi-modal treatment, survival, particularly in the metastatic setting, is still poor (1, 2). First-generation Receptor Tyrosine Kinase (RTK)-directed targeted therapies such as highly target-specific anti-IGF-1R antibodies have shown encouraging clinical effects, albeit in small subsets of patients, and mainly in ES (3–7). With both the advancement of our understanding in OS and ES biology and the development of novel RTK-targeted drugs, however, increases in clinical benefit (any effect between disease stabilization and complete remission) have been reported with more recently developed small molecule multi-RTK-inhibitors in around 50-80% of thus far molecularly unselected, heavily pre-treated OS and ES patients in prospective clinical trials (8, 9). These response rates are largely dominated by multi-RTK small molecule inhibitors that combine a strong anti-angiogenic component *via* inhibition of the vascular endothelial growth factor receptors (VEGFRs) with simultaneous inhibition of other key RTKs implicated in OS and ES progression, including the platelet-derived growth factor receptors (PDGFRs), c-KIT, fibroblast growth factor receptors (FGFRs), RET, and/or MET. Pazopanib (10), sorafenib (11), regorafenib (12, 13), anlotinib (14) and lenvatinib (15), which all have activity against VEGFR, PDGFR, c-KIT, FGFR and RET, and cabozantinib (16), which targets VEGFR, MET, AXL and RET, have all shown some clinical efficacy in OS and ES patients. In most instances, combination with chemotherapy showed efficacy as well (15, 17).

However, despite interesting clinical data, these agents have yet to become part of routine clinical care for OS and ES patients. Drug approval has proven to be difficult to obtain in sarcoma in general, but is particularly challenging for younger patients and those harboring bone sarcomas, not in the least because of their age, tumor subtype rarity, lack of actionable genomic aberrations, and sometimes challenging tumor response monitoring. Moreover, young patients often enter clinical trials after extensive previous polychemotherapy which may have affected organ function, and organ function reserve may be less than their young age suggests. Another major factor hampering progress for these patients is the overall hesitation of pharmaceutical companies to further develop their drugs for this rare patient population. One of the most well-known drugs in this field, pazopanib, is for example solely approved for use in advanced adult soft-tissue sarcoma (STS) (18). Although individual patient and retrospective reports also suggest efficacy of pazopanib in selected bone sarcoma patients, there has been no completed study that systematically tested its single-agent use prospectively in bone sarcoma patients. One phase II trial that was designed to evaluate the safety and anti-tumor efficacy of pazopanib in patients with unresectable, pulmonary metastatic OS was terminated prematurely prior to completion due to withdrawal of the sponsor’s financial support (19). Another phase II trial does highlight the anti-tumor potential of pazopanib combined with chemotherapy for OS treatment, with clinical benefit reported in 79% of patients, although this combination was associated with a high degree of toxicity (17). Other anti-angiogenic multi-RTK inhibitors, including sorafenib, regorafenib, anlotinib and lenvatinib, have been tested in larger bone sarcoma-focused trials, all showing potential effects, albeit mostly in terms of modestly extending the event-free survival (EFS). Although overall survival is yet to be improved, in this particularly difficult-to-treat patient population with a dismal prognosis, the general increase in EFS and reported individual tumor responses (including tumor shrinkage) are not to be ignored hints of activity that need to be taken forward. These results pave the road towards treatment refinements of such drugs, including pinpointing biomarkers to select those patients most likely to respond, and designing rational combinations to improve efficacy. It is at present however unclear which of these drugs, with largely overlapping molecular inhibition profiles, would work best for which patient, and treatment resistance almost uniformly occurs. As with any therapy, also these treatments are associated with side effects in a number of patients, so it is important to determine the toxicity of each of the drugs and to measure health-related quality of life in this often heavily pretreated population to better delineate the so-called net clinical benefit. Altogether, we provide a critical assessment and systemic comparison on the clinical outcomes and toxicity to the six most tested anti-angiogenic, multi-RTK-targeted drugs trialed in OS and ES to date, including pazopanib, sorafenib, regorafenib, anlotinib, lenvatinib and cabozantinib. We pay special attention to clinical response evaluations and drug-related toxicity in the context of OS and ES patients, and describe how future trials incorporating such drugs could be designed to ultimately improve response rates and decrease toxicity further.

## Methods

**Figure 1:**
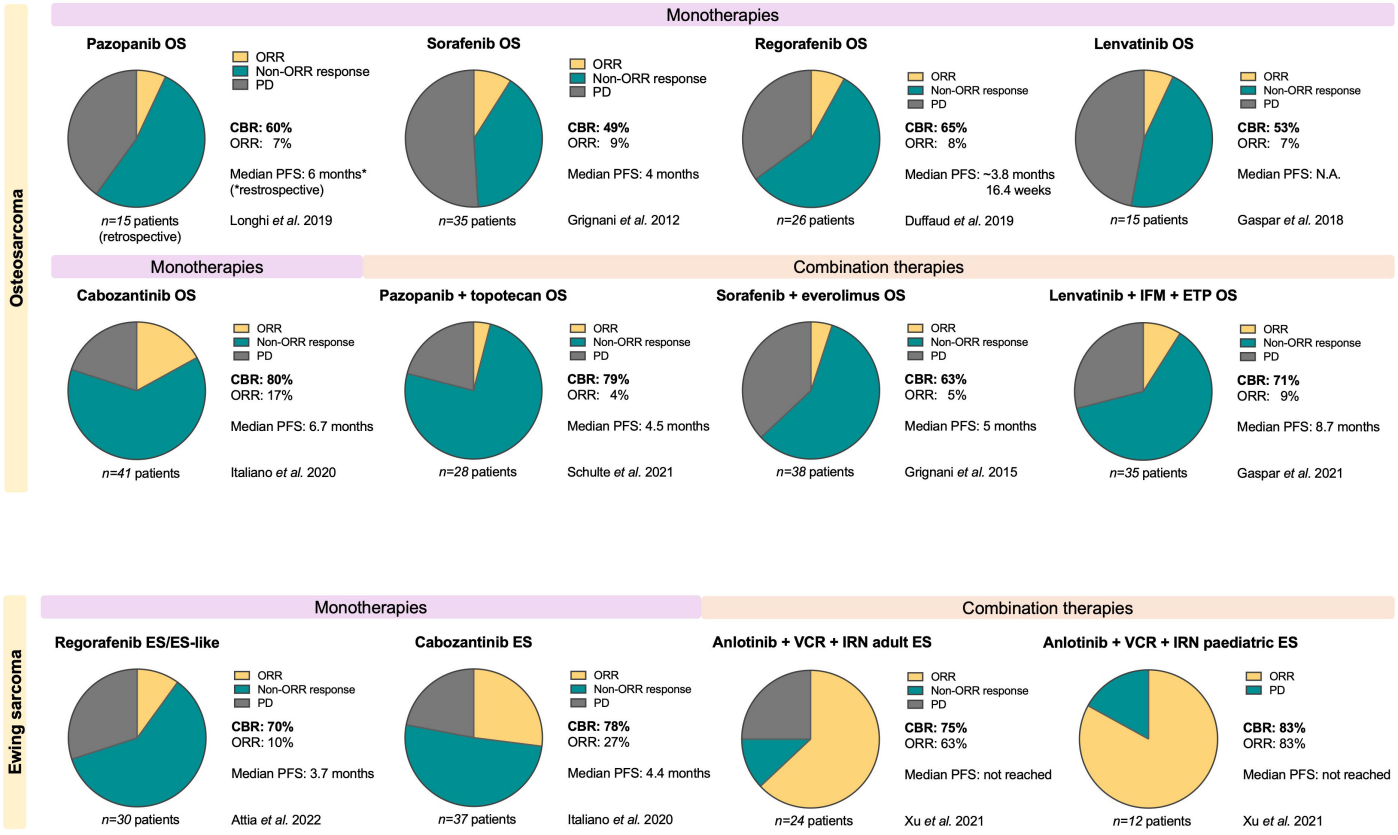
Overview of clinical responses to anti-angiogenic multi-RTK inhibitors in osteosarcoma and Ewing sarcoma patients. Only studies with at least 12 patients that also reported on non-objective response rates (non-ORR, defined as any non-objective response, including unconfirmed PR, Minor Response and Stable Disease (SD) to calculate Clinical Benefit Rate (CBR)) were included in this figure. ORR, Objective Response Rate (Complete Response (CR) + Partial Response (PR)); CBR, Clinical Benefit Rate (ORR + non-ORR); PFS, Progression-Free Survival; OS, osteosarcoma; ES, Ewing sarcoma; ETP, etoposide; IFM, ifosfamide; VCR, vincristine; IRN, irinotecan.

All studies reporting on the clinical efficacy of pazopanib, sorafenib, regorafenib, anlotinib, lenvatinib and cabozantinib in OS and ES patients up until May 1st 2022 were considered for inclusion in this review. This included case reports, retrospective studies and prospective clinical trials. For the main body of text and **Table 1**, all clinical reports were included. For **Figure 1**, only studies with at least 12 patients were included. In the majority of studies and throughout our review, tumor responses are reported according to RECIST criteria. They are categorized as a complete response (CR), partial response (PR), stable disease (SD) or progressive disease (PD). The objective response (OR) or objective response rate (ORR) is defined as the sum of the rates of CR and PR. The clinical benefit rate (CBR) is defined as the sum of ORR and SD. When reporting or comparing, to the best of our ability, the clinical efficacies of the different drugs discussed in this review, we take the best reported responses (CBR and ORR) for any period of time, and the median progression-free survival (PFS), into account.

**Table 1:** Overview of selected anti-angiogenic multi-receptor tyrosine kinase inhibitors in clinical trials in osteosarcoma and Ewing sarcoma.

Study/trial	Drugs	Inclusion tumour types	Age	Results	Reference
Pazopanib					
Case report	Pazopanib	Osteosarcoma	18, 21 and 23 years	Osteosarcoma *(n=3)* 2/3 SD, 1/3 PR CBR: 100%, ORR: 33% PFS: 6, 3* and >4 months	20
Retrospective	Pazopanib	Bone sarcomas (incl. OS, ES, CS and spindle cell/other)	18-62 years	Bone sarcoma (*n=19*, incl. 8 OS and 3 ES) 6/19 PR, 7/19 SD (subtype not specified) **CBR all: 68%, ORR all: 32%** (subtype not specified) All bone sarcomas median PFS: 5.45 months (95% CI 2.7–7.7) Osteosarcoma (*n=8*) 4/8 PR, SD unknown CBR: N/A**, ORR: 50% Ewing sarcoma (*n=3*) 1/3 PR, SD unknown CBR: N/A**, ORR: 33%	21
Retrospective	Pazopanib	Bone sarcomas (incl. OS and CS)	20-85 years	Bone sarcoma (*n=5*, incl. 3 OS and 2 CS) 2/5 SD (subtype not specified) **CBR: 40%, ORR: 0%** Median PFS: 10 months (95% CI 0.0-31.7)	22
Retrospective	Pazopanib	Osteosarcoma	11-69 years	Osteosarcoma (*n=15*): 1/15 PR, 8/15 SD, 6/15 PD (1 not evaluable) **CBR: 60%, ORR: 7%** Median PFS: 6 months (range 2–10)	23
Retrospective	Pazopanib	STS and bone sarcoma	14-85 years*	Osteosarcoma (*n=6*) 2/6 SD, 4/6 PD **CBR: 33%, ORR: 0%** PFS responders: 6 and 9 months; Ewing sarcoma (*n=3*) 2/3 SD, 1/3 PD **CBR: 67%, ORR: 0%** PFS responders: 6 and 13 months	10
Case report	Pazopanib	Osteosarcoma	19 years 25 years	Osteosarcoma (*n=2*) 1/2 reduction in all laesions, remission >2.5 years 1/2 SD for at least 3 months	24
Prospective	Pazopanib	Osteosarcoma	>18 years	Osteosarcoma (*n=7* evaluable) 4/7 SD >4 months, 3/7 PD **CBR: 57%, ORR: 0%**	19
Case report	Pazopanib	Ewing sarcoma	14 years	Near-complete remission 1 year	25
Case report	Pazopanib	Ewing sarcoma	24 years	PR up to 12 weeks	26
Case report	Pazopanib	Extra-osseous Ewing sarcoma	17 years	Response lasting >26 months	27
Case report	Pazopanib	Extra-osseous Ewing sarcoma	69 years	Tumour shrinkage for several weeks until patient passed away from another cause	28
Case report	Pazopanib	Extra-osseous Ewing sarcoma	62 years	70% reduction local lesion, SD liver metastasis for 2 months	29
Prospective	Pazopanib + Topotecan	Osteosarcoma	>18 years	Osteosarcoma (*n=28* evaluable) 1/28 PR, SD/PD not specified **CBR: 79%, ORR: 4%** Median PFS: 4.5 months	17
Prospective	Pazopanib + trametinib	STS (incl. ES)	22-77 years*	STS (*n=25*, incl. 4 ES) 4/4 ES no OR, 12/25 patients SD (subtype not specified) CBR: N/A**, ORR: 0%	30
Sorafenib					
Prospective	Sorafenib	Osteosarcoma	>14 years	Osteosarcoma (*n=35*) 3/35 PR, 2/35 minor response (<30% tumour shrinkage), 12/35 SD **CBR: 49%, ORR: 9%** Median PFS: 4 months (95% CI 2–5)	11
Retrospective	Sorafenib +/- chemotherapy	Bone sarcomas (incl. OS, ES and CS)	4–27 years	Osteosarcoma (*n=8*) Sorafenib mono (*n=3*): 2/3 PR, 1/3 SD Sorafenib + chemo (*n=5*): 4/5 PR, 1/5 SD Ewing sarcoma (*n=2*) Sorafenib mono (*n=1*): 1/1 PR Sorafenib + chemo (*n=1*): 1/1 PD Median PFS (OS cohort): 4 months (1.8–7.9)	31
Case report	Sorafenib	Osteosarcoma	7 years	PR for 51 months	32
Prospective	Sorafenib + everolimus	Osteosarcoma	>18 years	Osteosarcoma (*n=38*) 2/38 PR, 2/38 minor responce, 20/38 SD, 14/38 PD **CBR: 63%, ORR: 5%** Median PFS: 5 months (95% CI 2–7) 8 (21%) patients receiving treatment > 8 months	33
Pilot trial	Sorafenib + everolimus	Paediatric osteosarcoma	7–17 years	Osteosarcoma (*n=14*) Interim response data: mostly SD, rarely minor responses Median PFS (interim): 4.4 months	34
Prospective	Sorafenib + beva + chemo	Children/AYA solid tumours (incl. OS and ES)	1-22 years*	Children/AYA solid tumours (*n=24*, incl. 3 OS and 3 ES) OS (*n=3*): 2/3 SD (RECIST), 1/3 SD (non-RECIST). ES (*n=3*): 2/3 SD (RECIST), 1/3 SD (non-RECIST) **CBR (OS and ES): 100%, ORR (OS and ES): 0%**	35
Regorafenib					
Prospective	Regorafenib	Bone sarcomas (incl. OS and ES)	>10 years	Osteosarcoma (*n=38* evaluable; 12 placebo, 26 regorafenib) Regorafenib: 2/26 PR, 15/26 SD, 9/26 PD **CBR: 65%, ORR: 8%** Median PFS regorafenib: 16.4 weeks (95% CI 8.0–27.3) Median PFS placebo: 4.1 weeks (95% CI 3.0–5.7) Ewing sarcoma (*n=36* evaluable; 13 placebo, 23 regorafenib) Regorafenib: 5/23 PR, 18/22 SD or PD CBR: N/A** ORR: 22% Median PFS regorafenib: 11.4 weeks (95% CI 4.6-22.9) Median PFS placebo: 3.9 weeks (95% CI 3.3-7.3)	13 36
Prospective	Regorafenib	Bone sarcomas (incl. OS and ES)	18-76 years	Osteosarcoma **(** *n=42* evaluable: 20 placebo, 22 regorafenib) Regorafenib: 3/22 PR, 19/22 SD or PD CBR: N/A**, ORR: 14% Median PFS regorafenib: 3.6 months (95% CI 2.0-7.6) Median PFS placebo: 1.7 months (95% CI 1.2-1.8) Ewing sarcoma and ES-like (*n=30* regorafenib) 3/30 PR, 18/30 SD, 9/30 PD **CBR: 70%, ORR: 10%** Median PFS: 3.7 months (14.8 weeks; 95% CI 7.3 – 15.9 weeks)	12 37
Case report	Regorafenib	Osteosarcoma	17 years	Clinical response (shrinkage multiple tumour lesions) after 12 weeks of regorafenib	38
Anlotinib					
Prospective	Anlotinib	Bone sarcomas (incl. OS and ES)	14-68 years	Bone sarcomas (*n=42*, incl. 29 OS and 3 ES) **CBR all: 79%, ORR all 10%** PFS all: 5.26 months (95% CI 3.5–8.4) PFS OS: 4.83 months (95% CI 3.5–7.1)	14
Prospective	Anlotinib	Sarcoma (incl. OS)	8-79 years	Sarcomas (*n=31*, incl. 8 OS) **CBR all: 77%, ORR all: 29%** (no breakdown subtype-specific responses)	39
Retrospective	Anlotinib	STS and osteosarcoma	20 +/- 11 years (average)	Osteosarcoma (*n=13*) 1/13 PR, 3/13 SD **CBR: 31%, ORR: 8%** Median PFS: 2.7 +/- 1.6 months	40
Prospective	Anlotinib + chemotherapy	Ewing sarcoma	Adult and paediatric cohort	Adult cohort (*n=24*) 1/24 CR, 14/24 PR, 1/24 unconfirmed PR, 2/24 SD, 6/24 PD **CBR: 75%, ORR: 63%** Paediatric cohort (*n=12*) 4/12 CR, 6/12 PR, 2/12 PD **CBR: 83%, ORR: 83%**	41
Retrospective	Anlotinib + chemotherapy	STS (incl. ES)	15–69 years*	Ewing/PNET sarcoma (*n=3*) 2/3 PR, 1/3 SD **CBR: 100%, ORR: 67%**	42
Lenvatinib					
Prospective	Lenvatinib	Osteosarcoma	2-25 years	Osteosarcoma (*n=15*) 1/15 PR, 7/15 SD **CBR: 53%, ORR: 7%**	43
	Lenvatinib + chemotherapy	Osteosarcoma	2-25 years	Osteosarcoma (*n=32 and n=35* evaluable) 3/32 OR, 25/35 disease control (= CBR) **CBR: 71%, ORR: 9%** Median PFS: 8.7 months (95% CI 4.5–12.0)	15
Cabozantinib					
Prospective	Cabozantinib	Children/AYA solid tumours (incl. OS and ES)	4-18 years*	Children/AYA solid tumours (*n=41*, incl. 2 OS and 4 ES) OS (*n=2*): 2/2 PD. **CBR and ORR: 0%** ES (*n=4*): 1/4 SD, 3/4 PD. **CBR: 25%, ORR: 0%**	44
Prospective	Cabozantinib	Osteosarcoma and Ewing sarcoma	>12 years	Osteosarcoma (*n=41* evaluable best overall response) 7/41 PR, 26/41 SD, 8/41 PD **CBR: 80%, ORR: 17%** Median PFS: 6.7 months (95% CI 5.4–7.9) Ewing sarcoma (*n=37* evaluable best overall response) 10/37 PR, 19/37 SD, 8/37 PD **CBR: 78%, ORR: 27%** Median PFS ES: 4.4 months (95% CI 3.7–5.6)	16

Studies highlighted in grey are prospective studies. tumour types; STS, soft-tissue sarcoma; OS, osteosarcoma; ES, Ewing sarcoma; CS, chondrosarcoma. Drugs, beva, bevacizumab; chemo, chemotherapy. Responses; CR, Complete Response; PR, Partial response; SD, stable disease; PD, progressive disease; CBR, Clinical Benefit Rate (= ORR + SD, minor response and non-RECIST or non-confirmed PR), ORR, Objective Response Rate (= confirmed CR + PR), PFS, Progression-Free Survival.

* Age range of all patients included in respective study as age range of osteosarcoma or Ewing sarcoma patients was not specified.

** CBR or ORR could not be calculated as number of SDs or PRs was not further specified in the respective study.

CBR and ORR are shown in bold in all non-case report studies.

## Main drug targets of anti-angiogenic multi-RTK inhibitors

We selected six multi-target VEGF-receptor inhibitors, all with gross overlap in the additional RTK inhibition profile. However each inhibitor has a slightly different inhibition pattern with associating toxicity profiles. **Table 1** and **Figure 1** provide an overview of these six anti-angiogenic multi-RTK inhibitors clinically trialed in OS and ES, including a summary of clinical efficacy data. **Table 2** gives an overview of active studies of these drugs in OS and ES, as dated on Jan 24^th^ 2023. In addition, **Table 3** gives an overview of the targets and toxicity profiles of each of the anti-angiogenic multi-RTK inhibitors discussed in this review.

**Table 2:** Currently active clinical trials of selected anti-angiogenic multi-receptor tyrosine kinase inhibitors in osteosarcoma and Ewing sarcoma.

	OS	ES
**Pazopanib**	–	–
**Sorafenib**	–	• NCT01946529 (active, not recruiting)
**Regorafenib**	• NCT04803877 (SARC038) • NCT04055220 (REGOSTA) • NCT04698785 (REGOMAIN) • NCT02389244 (REGOBONE) • NCT05395741 (Regbone)	• NCT02389244 (REGOBONE) • NCT02085148 (active, not recruiting) • NCT05395741 (Regbone) • NCT04055220 (REGOSTA)
**Anlotinib**	–	• NCT03416517 (status unknown)
**Lenvatinib**	• NCT04154189 (active, not recruiting)	–
**Cabozantinib**	• NCT05019703 (TACOS) *(not yet recruiting)* • NCT02243605 *(active, not recruiting)* • NCT04661852 (active, not recruiting) • NCT05182164 (PEMBROCABOSARC) • NCT02867592 *(active, not recruiting)* • NCT05135975 • NCT05683197- NCT05691478 (not yet recruiting)	• NCT02243605 (active, not recruiting) • NCT04661852 (active, not recruiting) • NCT05182164 (PEMBROCABOSARC) • NCT02867592 (active, not recruiting) • NCT05135975

Data extracted from clinicaltrials.gov on 23 January 2023.

**Table 3:** Overview of toxicities related to selected anti-angiogenic multi-receptor tyrosine kinase inhibitors from prospective trials in osteosarcoma and Ewing sarcoma.

Treatment	Drug targets	Toxicities reported in prospective studies with sarcoma patients
**Pazopanib**	▪ VEGFR 1, 2, 3 ▪ PFDGR α and β ▪ FGFR 1 and 3 ▪ c-KIT ▪ LTK ▪ Lck ▪ M-CSF (c-Fms)	Pazopanib in OS patients ≥16 years ( *n=12* ): The most common SAEs (all grade 3) were elevated bilirubin (8%), elevated transaminases (8%), hypertension (8%) and decrease in ejection fraction (8%). No treatment-related grade 4 or 5 AE were reported. AE related drug discontinuation was reported in 17%. QoL was not reported (19). Pazopanib in combination with topotecan in sarcoma patients ≥18 years ( *n=153* ): The most common grade 3 toxicity was: decreased neutrophil count (46%), platelet count decrease (30%), hypertension (23%) and anaemia (20%). The most common grade 4 toxicity was also platelet count decrease (11%) and decreased neutrophil count (7%). Other common SAEs were hypertension (23%) and hyponatremia (11%). Three grade 5 treatment related toxicities were reported. AE related drug discontinuation was reported in 29% and dose reduction was necessary in 38%. QoL was not reported (17). Pazopanib in combination with trametinib in STS patients >18 years ( *n=25* ): The most common grade 3 toxicity included hypokalemia (12%), diarrhoea (12%), and thrombocytopenia (16%). Grade 4 toxicity was reported twice (anaemia and thrombocytopenia once each). No treatment related grade 5 toxicity was reported. Dose reduction of pazopanib was necessary in 20% of patients. QoL was not reported (30).
**Sorafenib**	▪ VEGFR 1 and 3 ▪ PDGFR β ▪ c-KIT ▪ RAF ▪ RET ▪ FLT3	Sorafenib in OS patients >14 years ( *n=35* ): 78% of patients had an adverse event. The most common grade 3 toxicity was hand-foot syndrome (9%). Four grade 4 toxicities were reported including: lipase elevation (3%), pneumothorax (3%) and CK elevation (6%). No treatment related grade 5 toxicity was reported. AE related drug discontinuation was reported in 46%. QoL was not reported (11). Sorafenib in combination with everolimus in OS patients ≥18 years ( *n=38* ): 100% of patients had AEs and 10% had a SAE. The most common grade 3 toxicity was hand-foot skin reaction (34%), diarrhoea (29%), infection (18%) and thrombocytopenia, oral mucositis and hypophosphatemia (18% each). The only grade 4 toxicity reported was lymphopenia in 8%. No treatment related grade 5 toxicity was reported. Dose modification or interruption due to AEs was necessary in 66% of patients. Permanent treatment discontinuation due to AE was reported in 5% of patients. QoL was not reported (33). Sorafenib in combination with bevacizumab and cyclophosphamide in patients with solid tumours <23 years ( *n=24* ): The most common SAEs were lymphopenia (71%), neutropenia (29%) and hypertension (17%). No treatment related grade 5 toxicity was reported. 25% of patients discontinued treatment due to toxicity. 29% of patients required dose reduction of sorafenib. QoL was not reported (35).
**Regorafenib**	▪ VEGFR 1, 2, 3 ▪ TIE2 ▪ PDGFR α and β ▪ FGFR 1 and 2 ▪ c-KIT ▪ RET ▪ RAF	Regorafenib in OS patients ≥ 10 years ( *n=29* ): 24% of patients had a SAE. The most common grade 3 toxicities were hypertension (24%), and hand-foot skin reaction, chest pain and fatigue (10% each). Two grade 4 toxicities were reported (one hypophosphatemia and one skin reaction). No treatment related grade 5 toxicity was reported. AE related dose reduction was necessary in 39% of patients. Most dose reductions were due to hand-foot syndrome (50%). Transient interruption due to toxicity was reported in 35% of patients. QoL was not reported (13). Regorafenib in OS ≥ 18 years ( *n=22* ): 64% of patients had a SAE. The most common grade 3 toxicity was hypertension (*n=3*). One grade 4 toxicity was reported (colonic perforation). No treatment related grade 5 toxicity was reported. Dose interruption was necessary in 59%. Dose reduction was reported in 55%. QoL was not reported (12).
**Anlotinib**	▪ VEGFR 2 and 3 ▪ PDGFR α and β ▪ FGFR 1, 2, 3, 4 ▪ c-KIT ▪ RET	Anlotinib in bone sarcoma patients ( *n=42* ): The most common SAEs were hypertension (19%), hypertriglyceridemia (10%), hand-foot syndrome (7%), and proteinuria (5%) (14). Anlotinib in sarcoma patients of all ages ( *n=31* ): One SAE (grade 3 hypertension) was reported in one patient (39). Anlotinib in combination with vincristine and irinotecan in ES patients of all ages ( *n=36* ): The most common SAEs were leukopenia (29%), neutropenia (24%), anaemia (9%) and diarrhea (4%). QoL was not reported (41).
**Lenvatinib**	▪ VEGFR 1, 2, 3 ▪ FGFR 1, 2, 3, 4 ▪ PDGFR α ▪ RET ▪ c-KIT	Lenvatinib combined with etoposide and ifosfamide in OS patients ≤25 years ( *n=35* ): 74% of patients had a treatment emergent SAE. The most common grade 3 and 4 toxicities were anaemia, thrombocytopenia and neutropenia. No grade 5 AE was reported. Treatment emergent AE led to dose reduction in 60-86% at various dosing schedules and treatment interruption was necessary in 54-71% of patients. Five patients withdrew from lenvatinib treatment because of treatment emergent AE. QoL was not reported (15).
**Cabozantinib**	▪ VEGFR 2 ▪ c-MET ▪ c-KIT ▪ FLT-3 ▪ AXL ▪ RET	Cabozantinib in ES and OS patients ≥12 years ( *n=90* ): 68% of patients had a SAE, no grade 5 AE was reported. Adverse events led to dose modification or definitive treatment discontinuation in 39% of patients. The most common grade 3 toxicities were: fatigue, oral mucositis and increase of liver enzymes. Four grade 4 toxicities were reported (hypomagnesemia (*n=1*), lipase increase (*n=2*) and neutropenia (*n=1*). QoL was not reported (16). Cabozantinib in children with various solid tumours ≤18 years ( *n=36* ): Six grade 4 toxicities and one grade 5 toxicity were reported, each occurring only once. Grade 3 toxicities included: hypertension (*n=3*), palmar-plantar erythrodysesthesia (*n=3*), diarrhea (*n=2*), increased ALT (*n=2*), proteinuria (*n=1*), fatigue (*n=1*), and weight loss (*n=1*). AE led to dose reduction between 0-36%, depending on the dose schedule, and treatment discontinuation was necessary in 0-18% of patients, depending on the dose schedule. QoL was not reported (44).

VEGFR, Vascular Endothelial Growth Factor Receptor; PDGFR, Platelet-Derived Growth Factor Receptor; FGFR, Fibroblast Growth Factor Receptor; LTK, Leukocyte Receptor Tyrosine Kinase; Lck, Lymphocyte-specific protein tyrosine kinase; M-CSF, Macrophage Colony-Stimulating Factor; RAF, Rapidly Accelerated Fibrosarcoma; AE, Adverse Events; SAE, Serious Adverse Events (defined as, grade ≥3 toxicity); QoL, Quality of Life.

## Pazopanib

Pazopanib is a well-known drug in the family of anti-angiogenic multi-RTK-targeted drugs. It is the only targeted agent approved for second line systemic treatment in adults with advanced and metastatic soft-tissue sarcomas (but not for gastrointestinal stromal tumors (GIST) and adipocytic sarcomas). The approval was based on a significant gain in PFS against placebo in the pivotal phase III PALETTE study, but without evidence of a benefit on overall survival (18). Pazopanib is not approved for pediatric sarcoma patients, nor for bone sarcoma patients. Its potential efficacy in OS and ES patients was demonstrated, however mostly not in a clinical trial setting, but rather in retrospective single-center studies or case reports. One of the first reports, in 2014, demonstrated clinical activity in three consecutive, progressive metastatic OS patients, including SD in two patients (lasting between 3 and 6 months, one tumor showing cystic changes) and marked tumor shrinkage in one patient lasting for at least four months. Responses were accompanied with improvement of general condition and, in one case, pain relief (20). A recent retrospective analysis examined the clinical experience of pazopanib in the treatment of 19 bone sarcoma patients, including 8 OS and 3 ES. Clinical benefit was reported in 68% (13/19) of bone sarcoma patients with a median PFS of 5.45 months across all included bone sarcoma subtypes (95% CI 2.7 – 7.7) (21). This study highlighted a PR in 4/8 OS patients and 1/3 ES patients. The subtype-specific CBRs could however not be determined as no information was provided on the bone sarcoma subtypes of those patients that experienced an SD, nor was a subtype-specific PFS reported. Another unicentric retrospective analysis investigating the efficacy of pazopanib in five metastatic, adult bone sarcomas, consisting of two chondrosarcoma and three OS patients, reported SD in two of these patients (subtype not specified), resulting in a CBR of 40% and a median PFS of 10 months (95% CI 0.0-31.7) (22). A combined report from two large sarcoma reference centers reported 1 PR and 8 SD out of 15 OS patients. The CBR and ORR consequently were 60% and 7%, respectively, with a median PFS of 6 months (range 2–10) (23). Another retrospective analysis on pazopanib monotherapy in STS and bone sarcomas reported SD in 2/6 OS (6 and 9 months) and 2/3 ES patients (6 and 13 months) (10). In two relapsed OS patients, an impressive reduction in all lesions was reported in a 19-year-old patient treated with pazopanib and radiotherapy, who remained on treatment and in remission for >2.5 years. Although the contribution of radiation could not be determined in this case, historically OS is not considered to be a radiosensitive tumor. The other, a 25-year-old, achieved SD for the 3 months on pazopanib treatment, and had rapid disease progression after discontinued treatment due to gastrointestinal toxicity (24). Finally, in 7 evaluable patients with OS metastatic to the lung, SD >4 months was reported in 4 patients, while 3 had PD (19). A phase II study of pazopanib combined with oral topotecan tested in among other patients (18 years or older) with metastatic OS further reported a CBR of 79% but only an ORR of 4% in the 28 included OS patients, with a median PFS of 4.5 months and overall survival of 11.1 months. The CBR in the OS cohort was higher than the CBR observed in the STS (71%) and liposarcoma (44%) cohorts, making this potentially an interesting combination for OS patients (17). The adverse events associated with this trial were however higher than in the previously conducted phase I trial. As, at least from a retrospective analysis, pazopanib therapy alone yielded a median PFS of 6 months, a prospective randomized controlled study is warranted to dissect the true added value of this combination (23). This is particularly important given the reported adverse events, where refinements in both the treatment protocol plus selection of patients most likely to benefit are needed if this particular combination is further pursued (45). It will be important to avoid unnecessary treatment in young patients, despite the general tendency to treat young cancer patients actively for long in the palliative setting (46).

Although the use of pazopanib in ES patients is less studied, in addition to the two SDs reported above, there have been reports of individual responders. A remarkable response was noted in a 24-year-old metastatic and chemotherapy refractory ES patient, showing a PR for 12 weeks (26). Clinical efficacy of pazopanib was further reported in three patients with extra-osseous ES, including a response lasting over 26 months in a 17-year-old patient (27), tumor shrinkage during treatment in a 69-year-old patient for several weeks until the patient passed away from another cause (28), and marked reduction (70%) in local lesion and liver metastasis disease stabilization up to 2 months in a 62-year-old patient (29).

To accurately assess the clinical utility of pazopanib in bone sarcoma patients, larger, prospective and randomized studies are needed as publication bias most probably plays a role in these small numbers. All studies in OS and ES so far, however, do suggest some effect which requires further research of pazopanib, or pazopanib combination treatments, in treating these malignancies, preferably not at an end stage of disease.

There has been some work on reporting clinical efficacy of other, non-chemotherapy-based, pazopanib combination treatments in bone sarcoma patients, although the exact effects of these treatments on OS and ES patients remains elusive. A retrospective study evaluated the safety and efficacy of pazopanib plus vorinostat (HDAC-inhibitor), everolimus (mTOR-inhibitor), lapatinib or trastuzumab (HER2-inhibitors), and trametinib (MEK-inhibitor) in patients with advanced sarcoma, including bone sarcomas (11 ES, 5 OS, and 2 chondrosarcomas). Of all evaluable 43 patients, 4 PRs and 16 SDs were reported, although subtypes were not further specified. The median PFS was 8.9 weeks for bone sarcoma patients (versus 14.5 weeks for STS), although no details were given which combinations these patients received. As, not unexpected, the median PFS between the different combination treatments also differed, and again no details were given on which patients received which treatments and no biomarkers predictive of response were included, no real conclusions can be drawn on the efficacies of these combinations in OS and ES patients (47). This study did demonstrate overall safety of these pazopanib combinations and no unexpected toxicities. A study evaluating the combination of pazopanib and trametinib in advanced STS reported no objective response in the four included ES patients. When comparing the tumor molecular profile of one of the non-responding ES patients to another patient treated previously in their clinic that did respond to pazopanib, they found that *FGFR3*, *FGFR4* and *FLT4* (VEGFR3) genes were amplified only in the responder (30). SD was reported in 12 out of the 25 patients included in total, although also here no further details on histology were provided.

## Sorafenib

Although sorafenib is not approved for use in sarcomas, we note its approval for adult refractory or advanced desmoid tumors, a locally aggressive non-metastatic soft tissue tumor (48). Sorafenib was one of the first anti-angiogenic, multi-RTK inhibitors tested in a prospective clinical trial setting in OS. In 2011, a phase II trial of sorafenib in relapsed and unresectable high-grade OS patients >14 years after failure of standard multimodal therapy reported 3 PRs, 2 minor responses (MR; <30% tumor shrinkage) and 12 SDs in 35 patients, totaling a CBR of 49% and ORR of 9% at any time as MRs are not recognized in the RECIST criteria. The study primary endpoint measure, 6-month PFS, was 29%. Median PFS was 4 (95% CI 2–5) months. The youngest included patient was 15 years, and in total 7 patients <18 years were included (11). In another, retrospective, study, twelve patients with refractory bone tumors (8 OS, 2 ES and 2 chondrosarcoma) received treatment with sorafenib, including four patients under the age of 15 (range 4.1–27.9 years). Unfortunately, study results are difficult to interpret as seven of these patients were treated with sorafenib in combination with chemotherapy. Of the 5 patients treated solely with sorafenib, four experienced PR and one had SD (one ES, three OS and one chondrosarcoma patient). Duration of treatment with monotherapy sorafenib ranged from 50-299 days, and one patient had to discontinue after 13 days due to unacceptable skin toxicity (31). A case report further reported a prolonged PR in a 7-year-old OS patient treated with sorafenib for 51 months. The sorafenib-naïve tumor harbored a *PDGFRA* D846V mutation that was not identified in the relapse specimen, thereby providing both a biomarker for response and resistance to be studied in similar cases (32).

When sorafenib was combined with everolimus, 2 PRs, 2 MRs, and 20 SDs were reported in 38 adult OS patients, resulting in a CBR of 63% and ORR (RECIST) of 5%. The median PFS was 5 months (95% CI 2–7), with eight (21%) patients receiving sorafenib and everolimus for 8 months or more. The 6-month PFS was 45%, which is encouraging, but did not reach the study’s predefined strict endpoint of 50% (33). The toxicity was generally manageable with short dose reductions and confirmed treatment feasibility. Immunohistochemical expression of pERK1/2 and pRPS6 was associated with a better response to study drugs. This same combination was trialed in 14 pediatric and AYA (adolescent and young adult) OS patients (median age 14 years, range 7 – 17 years). Interim response data included mostly SD. Median PFS was 4.4 months (34). A recent phase I study examining the efficacy of sorafenib, bevacizumab and chemotherapy (low-dose cyclophosphamide) in children and young adults with refractory or recurrent solid tumors reported SD in 2/3 OS and 2/3 ES patients, and a non-RECIST SD in the other OS and ES patients (35).

## Regorafenib

Regorafenib has in two clinical trials consistently shown to increase PFS, but not overall survival, as a single agent in OS patients. The REGOBONE study, a randomized phase II double-blind placebo-controlled clinical trial, reported clinical benefit at 8 weeks in 65% (17/26) of the 26 evaluable progressive metastatic OS patients treated with single agent regorafenib, of which 8% had an ORR (2/26), versus solely progressive disease at 8 weeks in the placebo group (*n=12*). This translated to an EFS benefit for OS patients treated with regorafenib of 3.8 months (16.4 weeks; 95% CI 8.0–27.3 weeks) versus 0.9 months (4.1 weeks; 95% CI 3.0–5.7 weeks) in the placebo control arm. Ten patients in the placebo group crossed over to receive open-label regorafenib after centrally confirmed disease progression (13). The SARC024 trial, a multi strata sarcoma randomized double-blind placebo-controlled phase II study, also tested efficacy of regorafenib in 22 patients with metastatic OS. Although the exact CBR could not be calculated as SD was not reported as an outcome measure, the ORR and PFS were largely comparable to those of the REGOBONE trial. The median PFS was 3.6 months (95% CI 2.0-7.6 months) in the regorafenib arm versus 1.7 months (95% CI 1.2-1.8 months) in the placebo arm (p=0.017), with an ORR of 14% (3/22) versus none respectively (12). It is encouraging that these two separate placebo-controlled randomized clinical trials show the same level of meaningful efficacy of single agent regorafenib in metastatic OS patients, particularly when reflecting on the reported EFS of only one month of patients treated with placebo, reflecting the aggressiveness of this disease. In both trials, the overall toxicity and safety of regorafenib was reported to be acceptable, and adverse events were generally manageable with dose reductions. Of course, in these patients, one would ultimately aim for overall survival benefit, and the moment of introducing these drugs in the standard treatment schedule could be subject to further study. As a result of these studies, the ESMO–EURACAN–GENTURIS–ERN PaedCan Clinical Practice Guideline referred to regorafenib in their guidelines of 2021 as potential second line treatment associated with evidence of activity in relapsed or metastatic OS. Regorafenib is now also listed as a second line therapy for recurrent OS in the National Comprehensive Cancer Network (NCCN) Guidelines of the United States. However, as regorafenib is not approved by the European Medicines Agency (EMA) for sarcomas other than (later line) advanced/metastatic gastrointestinal stromal tumor (GIST), it is not reimbursed and cannot be prescribed as standard practice in European countries for OS (49). Furthermore, the major pediatric oncology groups worldwide have yet to study or endorse regorafenib for progressive OS. The youngest patient included in REGOBONE was 21 years of age (despite recruitments open to >10 years), and SARC024 included adult patients only. The potential of regorafenib for adolescent OS patients was highlighted in a recent report, where a 17-year-old patient with progressive metastatic OS with pulmonary nodules achieved a clinical response after 12 weeks of regorafenib, with tumor shrinkage in multiple lesions (38). Side effects were manageable and the patient continued to receive treatment at the time of publication of the report.

The data on the SARC024 trial on the ES and ES-like cohort further showed a CBR and ORR of regorafenib monotherapy in 70% (21/30) and 10% (3/30) of these patients. The median PFS was 3.7 months (37). Interim data from the ES cohort of the REGOBONE trial reported that 57% (13/23) of patients were non-progressive at 8 weeks *vs*. 8% (1/13) of patients in the placebo control arm. The ORR was 22% (5/23) and the median PFS for regorafenib was 11.4 weeks *vs.* 3.9 weeks in the control arm (36). CBR could not be calculated as SD was not reported as an outcome measure, and toxicity was reported to be moderate. These results exemplify that also for ES patients regorafenib may be an option in the advanced setting, although also here improvements with for example combinations therapies are required to further improve the response rate, and ultimately overall survival. It is interesting to note that regorafenib is currently still the topic of investigation in a number of clinical trials in OS and ES (**Table 2**).

## Anlotinib

Anlotinib has, both as single agent and in combination with chemotherapy, repeatedly shown meaningful clinical efficacy in bone sarcoma patients. Anlotinib has been approved in China as a second-line treatment for STS after anthracyclines. In addition, anlotinib is currently investigated in several STS subtypes in the phase III APROMISS study executed in China, Italy, USA, UK and Spain (NCT03016819). Of the 42 evaluable relapsed or metastatic bone sarcoma patients (including 29 OS and 3 ES patients) treated with single agent anlotinib in an ongoing Phase II trial, disease control was observed in 79% of patients, of which 10% achieved an OR. Median PFS was 5.3 months (95%CI 3.5-8.4) across the different subtypes (14). Another phase IV clinical trial that tested the efficacy of anlotinib in sarcoma patients after failure of first-line chemotherapy, included 5 patients with OS (total study population 40 patients). A disease control rate of 77.5% and an ORR of 15% were observed, with a median PFS of 7 months for the total group. For the OS patients, the median PFS was however only 2.9 months (50). A retrospective review examining the safety and efficacy of anlotinib monotherapy in advanced sarcoma patients, including advanced OS, showed disease control in 31% (4/13) of included OS patients, of which 8% (1/13) reflected an OR. The median PFS was however only 2.7 months (40). Anlotinib has also been tested in combination with chemotherapy. In a retrospective trial including 3 adult ES patients, anlotinib in combination with chemotherapy (no specification which chemotherapy treatment regimen was used in the ES patients) showed 2 PR and 1 SD with a manageable toxicity profile without toxicity leading to treatment discontinuation (42). Although interesting, the numbers of treated patients is low, and it is difficult to estimate the expected clinical benefit or toxicity of the tested combination in the absence of full disclosure of the included chemotherapies. A phase Ib-II study evaluated the combination of anlotinib with vincristine and irinotecan and demonstrated an acceptable toxicity profile. Patients were divided into children (<16 years) and adults (≥ 16 years), with an ORR at 12 weeks of 83.3% (4 CR, 6 PR in a total study group of 12 evaluable patients) and 62.5% (1 CR, 14 PR, 2 SD in a total study group of 23 evaluable patients), respectively (41). The additional value of anlotinib to this chemotherapy regimen however needs further evaluation. Altogether, currently available data indicates that anlotinib, with or without chemotherapy, is a noteworthy targeted drug for the treatment of both OS and ES patients. The fact that even CRs were achieved in a substantial proportion of young ES patients when anlotinib was combined with vincristine and irinotecan with overall manageable toxicity, supports further testing of this particular treatment regimen, although it is at present difficult to determine to what extent these results are attributed to anlotinib. A randomized trial, including a vincristine and irinotecan chemotherapy only arm, to objectively assess the added value of anlotinib would be most welcome. The early clinical data of single agent anlotinib efficacy after failure of first-line therapy observed in sarcoma is another promising lead, as it suggests successful use of a targeted treatment much earlier on in the treatment plan than is routinely the case. If we can avoid having to wait for multiple lines of chemotherapy to fail before a novel treatment can be introduced, this will spare these often-young patients significant toxic side-effects. It also increases the chance for a better tolerability of the newly introduced drug.

## Lenvatinib

Lenvatinib is the latest drug added to the treatment armamentarium of VEGFR/PDGFR/FGFR/RET-inhibitors trialed in OS. As a single agent, lenvatinib showed some efficacy in OS patients in an Phase I/II trial including children and adolescents with refractory and relapsed solid malignancies and young adults with OS. Disease control and ORR were reported in 53% (8/15) and 7% (1/15) of OS patients, respectively, with a median number of cycles received of 3 (range 1-9) (43). Interim data of this same trial also showed inclusion of 5 rhabdomyosarcoma (RMS) and 4 ES patients so far, and disease control was reported in 43% (10/23) of patients with non-OS tumors. These were all SDs, and no ORR was reported in those subtypes thus far (51). Combination of lenvatinib with the chemotherapeutics etoposide and ifosfamide also showed promising anti-tumor activity in OS patients, with disease control reported in 71% (25/35 SD or PR) of evaluable treated OS patients, of which 9% (3/32) involved an OR (PR) (15). The median PFS was 8.7 months. This combination had a manageable safety profile with promising preliminary evidence of efficacy, and its efficacy is currently being compared to ifosfamide and etoposide without the addition of lenvatinib in children, adolescents and young adults with relapsed or refractory OS (NCT04154189). This is an important and valuable step into truly dissecting the added effects of lenvatinib to these types of chemotherapies compared to these chemotherapies alone. Another ongoing Phase I/II trial is testing the efficacy of lenvatinib in combination with the mTOR inhibitor everolimus in recurrent and refractory pediatric solid tumors, including ES and RMS patients (NCT03245151). Interim data on the phase I dose escalation study shows that 22% (2/9) of the evaluable patients so far (tumor types not specified) had SD as best overall response (52). Enrolment in the phase II part of the study, which specifically includes ES and RMS patients, is ongoing (NCT03245151). In other recurrent/refractory advanced solid tumors, the addition of trametinib to the combination of everolimus and lenvatinib will be tested, which, if deemed safe, could be a line of future investigation for OS and ES patients too (NCT04803318).

## Cabozantinib

Cabozantinib is unique and different from the above-mentioned angiogenesis-centered multi-RTK drugs regarding its drug target profile. Beyond strongly inhibiting angiogenesis like the others, cabozantinib’s additional drug profile is not centered around targeting PDGFR, FGFR and KIT, but instead is directed against MET, AXL and RET (53). As a single agent in a Phase I trial in children and adolescents with recurrent or refractory solid tumors, including 4 ES and 2 OS patients treated at different doses, prolonged SD was reported in 1/4 ES patients. This patient continued treatment till at least cycle 13 (data cut-off) on the 40 mg/m2 per day dosing schedule (44). No responses were reported in the other ES and OS patients. In a phase II trial at this same dosing schedule, cabozantinib showed encouraging efficacy in heavily pretreated OS and ES patients, with a median follow-up of 31 months. Objective responses were observed in 27% (10/37 PR) and 17% (7/41 PR) of RECIST-evaluable ES and OS patients, respectively. Clinical benefit was observed in 78% of ES patients and 80% of OS patients, with a median PFS of 4.4 and 6.7 months in ES and OS patients, respectively. The six month non-progression rate was 25.6% (ES patients) and 33.3% (OS patients) (16). Another Phase II trial in advanced non-breast and non-prostate malignancies, including 14 sarcoma patients (3 OS and 1 ES), reported preliminary efficacy (decrease in tumor size >10%) in 5 out of 8 evaluable, not further specified sarcoma patients (53). As cabozantinib was reported to be generally well-tolerated, it represents a new therapeutic option for these patients and warrants further investigation. We do note that in clinical practice, as with many other anti-angiogenic multi-RTK targeting drugs, dose reductions or dose interruptions are often required to achieve the overall well-tolerability profile. Refining the group of patients most likely to respond by incorporating molecular response markers to the currently largely histotype-guided clinical trials will be an important step forwards. As with other multi-kinase inhibitors, combinations with for example standard-of-care chemotherapies should be investigated. In patients with heavily pretreated relapsed leiomyosarcoma for example, the addition of cabozantinib to temozolomide and bevacizumab resulted in synergistic effects as 100% (6/6) patients treated with the three drugs showed a clinical benefit, of which 50% had an OR (2/6 CR and 2/6 PR). As a reference, the combination of temozolomide and bevacizumab without cabozantinib resulted in a CBR of 78%, with an ORR of 35% in these patients (2/14 CR and 3/14 PR) (54). It must however be taken into account that patient numbers were small in these studies and larger, randomized prospective trials are required to assess the exact benefit of adding cabozantinib to other drugs. There are currently several trials actively investigating the efficacy of cabozantinib in OS and ES patients, either alone or in combination with chemotherapy or immunotherapy (**Table 2**). This includes a phase II/III trial aimed to test the addition of cabozantinib to chemotherapy in patients with newly diagnosed OS (NCT05691478, not yet recruiting), thereby representing an important study in this field.

## Toxicity of anti-angiogenic multi-RTK inhibitors

Although in most studies the anti-angiogenic multi-RTK inhibitors described in this review showed an overall manageable toxicity profile, there are a number of important considerations to take into account. The wording ‘manageable toxicity’ is a widely used expression, but without quality-of-life assessments alongside clinical drug studies, one should be careful to mention the impacts of toxicity for a patient as manageable. In clinical practice, patients often need dose reductions of multi-RTK inhibitors due to toxicities diminishing daily quality of life. As the inhibitors mentioned in this review largely share the receptors they target, overlapping toxicities are seen. The most common serious adverse events are mainly fatigue (11-23%) and hypertension (7-23%). Palmar-plantar erythrodysaesthesia as serious adverse event is most commonly observed with sorafenib, anlotinib, cabozantinib and regorafenib treatment (8-20%). Other common adverse events seen across all treatments are diarrhea (35-58%), nausea (27-54%), and weight loss (22-48%). Treatment-specific frequent adverse events have also been reported. These include hair color depigmentation with pazopanib, bone marrow toxicity with lenvatinib and hypertriglyceridemia with anlotinib (18, 55–57). Adverse events, particularly with a chronic or serious character, often make dose reduction and treatment interruption necessary. **Table 3** summarizes the toxicity reported with each of the anti-angiogenic multi-RTK inhibitors described in this review specifically in OS and ES patients. This includes data from single agent anti-angiogenic multi-RTK inhibitor trials, as well as tested combinations including for example chemotherapy. The results in this **Table 3** illustrate that grade 3 and 4 adverse events frequently occur (up to 74%), and even grade 5 adverse events are reported. Particularly when pursuing or designing drug combinations for future trials, it will be important to pay careful attention to the expected drug-related toxicities. Several side effects, including renal, hepatic or bone marrow dysfunctions, but also skin toxicities and fatigue, are shared between chemotherapy and anti-angiogenic multi-RTK inhibitors. The dosing of the different drugs as well as their treatment schedules will hence need careful consideration. From a pharmacological point of view, one approach that could aid in both improving drug efficacy plus decrease drug-related toxicities, is to individualize the drug dose for each patient based on the actual measured drug levels (therapeutic drug monitoring), which has been trialed in single agent clinical settings (58). A point to pay special attention to when pursuing combination treatments, is the drug schedule, due to potential interferences. Drug interaction studies are essential, as exemplified by Hamberg et al., as this study showed that bolus ifosfamide infusion was too toxic to be combined with pazopanib, whereas continuous ifosfamide infusion in combination with pazopanib appeared to be safe. This study also showed that pazopanib exposure declined with the addition of ifosfamide, which could potentially influence its effect, although, at least in this study, pazopanib still exerted biological activity as demonstrated by a dose-dependent increase in PIGF and VEGF-A, the latter being the main ligand of VEGFR2, and a concurrent decline in soluble VEGFR2 (sVEGFR2) (59). Another study reported that when pazopanib was combined to topotecan, pazopanib substantially increased the exposure of oral topotecan, which may contribute to higher toxicity (45). As in clinical practice, novel drugs are often added to standard-of-care chemotherapeutic backbones, we need to pay special attention to their specific toxicity profiles. Regorafenib for example is associated with an increased risk of hepatotoxicity, which is also a major side effect of methothrexate, a cytotoxic agent commonly included in OS treatment. Furthermore, anti-angiogenic compounds pose a risk for developing cardiotoxicity, which is a known side effect of doxorubicin too, another cytotoxic agent used for OS treatment. It will be important to pursue rational drug combinations that will not only result in therapeutic enhancements, but are also tolerable. Randomized trials are important to tease out the exact added value of the combination therapy versus the anti-angiogenic multi-RTK inhibitor or chemotherapy alone, as well as to critically assess the associated toxicity profiles. Unfortunately, none of the prospective studies have reported on health-related quality of life data, therefore it remains to be determined what impact toxicities had on the health-related quality of life of OS and ES patients. Furthermore, underreporting of adverse events by oncologists is a well-known phenomenon, particularly outside the context of clinical trials (60).

Another aspect that deserves further consideration, is the timing of the introduction of anti-angiogenic multi-RTK inhibitors. In the vast majority of trials reported to date, novel agents are introduced after failure to multiple lines of cytotoxic therapies. It may hence not come as a surprise that these patients experience tolerability issues, even with drugs that are considered generally more targeted and more tolerable than chemotherapy. Introduction of novel compounds such as anti-angiogenic multi-RTK inhibitors earlier in the disease might come with a better tolerability profile, which would be an interesting subject for further study. Effects on fertility are largely unknown as most of these drugs have been involved in later line clinical studies after extensive chemotherapy schedules. When considering to bring these drugs to an earlier moment in the treatment paradigm, certainly in younger patients, this is, next to the effects of chemotherapy, a factor of consideration.

## Improving response rates – molecular response biomarkers

Although the anti-angiogenic multi-RTK inhibitors described in this review showed some efficacy across unselected OS and ES patient populations, the median PFS of all patients included per study was increased only modestly. Individual outliers have however been reported for each of those drugs, including continued disease stabilization or OR for over a year with for example pazopanib (10, 24). Together with the reported toxicity in some patients, this underlines a need for biomarker identification to select those patients most likely to respond, and prevent treatment in those that will likely not benefit. Early exploratory data in some of the clinical trials described in this review, or data derived from case reports, illustrate that incorporation of molecular biomarkers can indeed further refine the group of patients most likely to respond. Molecular response biomarkers have been investigated retrospectively in a number of the clinical trials, including phosphorylated-ERK1/2 (p-ERK1/2) and pRPS6, which were associated with a better response to the combination of sorafenib and everolimus (33), a *PDGFRA* D846V mutation that was linked to long term efficacy of sorafenib monotherapy (32), and amplification of *FGFR3*, *FGFR4* and *FLT4* (VEGFR3) genes which was detected only in the pazopanib-responsive ES patient (30). In the cabozantinib trial, prespecified, exploratory analyses of potential plasma biomarkers of cabozantinib response were performed by plasma analysis of VEGF-A, hepatocyte growth factor (HGF), soluble VEGFR2 (sVEGFR2), and soluble MET (sMET) in OS and ES patients (16). Although in the 39 eligible and assessable ES patients no associations with outcome were found, there was an association between low VEGF-A concentrations and improved overall survival, and between high sMET concentrations and improved PFS among the 42 eligible and assessable OS patients. More systematic research into defining the best biomarkers for response to each of the drugs however is required, also because case reports tend to include reporting of positive drug responses, and larger sample sizes are required to move away from anecdotal evidence.

## Novel predictive biomarker avenues: Target overexpression and activation

Kinase overexpression and/or activation signatures are becoming increasingly recognized as potentially actionable driver targets in OS and ES tumors. In various pediatric and AYA sarcoma models, including OS and ES, drug target/ligand overexpression and activation signatures could be linked to kinase inhibitor sensitivity, including examples of potential correlations for anti-angiogenic multi-RTK inhibitors (9, 61–66). In preclinical OS models, sorafenib treatment for example stabilized growth in an OS patient-derived xenograft (PDX) model with high VEGFR2 and VEGFA expression, whereas a non-VEGFA-amplified OS model did not respond (64). Out of a panel of 5 ES cell lines, the one ES cell line that had the lowest MET mRNA expression levels was the relatively most resistant one to cabozantinib or crizotinib (ALK/MET-inhibitor) (62). As a clinical example in another sarcoma subtype, we note a recent case study reporting on the efficacy of pazopanib in two patients with intimal sarcoma of the pulmonary artery. The patient that presented with moderate to strong expression of PDGFR-α and -β did derive some benefit from pazopanib (PFS 5.8 months), whereas the patient presenting with weak expression of PDGFR-α and -β had rapid disease progression (PFS 1.1 months) (67). Although more research in more models, or ideally patients, is required to truly test the predictive value of such overexpression markers, it does present a promising avenue for future research. In this context, it is interesting to note that expression of the main targets of the drugs described in this review, VEGFR, PDGFR, FGFR, c-KIT, RET, MET and AXL, is reasonably well-documented for OS and ES patients, with the vast majority of patients presenting some level of protein expression (4, 68). Current reports further suggest that 30-60% of OS or ES patients present with high protein expression levels of either one of the RTK-targets itself, or its main ligand. In most instances these represent the patients with the poorest prognosis, suggesting that in such instances the overexpression levels might indeed represent an actionable tumor driver signature. Here we exemplify high VEGFR2 (significantly poorer survival), VEGF (significantly poorer PFS), high PDGFRA (trend towards poorer outcome) and PDGF-AA (significantly poorer outcome) expression in 60%, 74%, 47% and 35% of OS patients, respectively (69–71). High MET, membranous MET, and high AXL expression characterized 30%, 34% and 36% of ES patient samples, where membranous MET and high AXL expression significantly correlated with a poor survival (62, 72).

As it is well known that activation of the drug targets and associated pathways is required to mediate sensitivity to targeted drugs, another approach could be to directly investigate drug target activation levels (9). In OS cell lines and patient-derived models for example, PDGFR and FGFR1/2 (pazopanib, sorafenib, regorafenib, anlotinib and lenvatinib targets), and AXL, MET and RET (cabozantinib targets) were among the proteins found to be recurrently activated. RET was found to be recurrently affected in ES (9). In individual cases, higher levels of for example pPDGFR were also demonstrated in ES, and also within the group of OS there were differences in drug target activation levels (61, 63). One of the main advantages of phosphoproteomics investigations over gene overexpression signatures, is that pathway activity can in many instances act as a more specific predictive or response biomarker compared to target expression levels (9). A particular challenge in the clinical setting however, is maintaining specimen integrity to allow the generation of high-quality phosphoproteomics data. One practical approach could be to screen patients for a selection of activated drug targets with for example a targeted phospho-protein array, immunohistochemistry or Western Blot, which is all feasible with a relatively small amount of tumor sample. For target activation levels as well as target/ligand overexpression signatures, it would be valuable to conduct more research into testing their capacity to predict response to anti-angiogenic multi-RTK inhibitors in OS and ES.

## Perspectives

All anti-angiogenic multi-RTK inhibitors described in this review tested in OS and ES patients to date, have shown reports of some clinical benefit, even as a single agent. As a single agent, we note that the ORR and CBR of cabozantinib exceeded those of the other drugs for both OS and ES. For ES patients, the CABONE trial noted that the majority of ES patients treated experienced tumor shrinkage, and the ORR was among the highest to have been observed with a tyrosine kinase inhibitor targeting the VEGFR2 pathway in solid tumors, with the exception of renal cell carcinoma, which has a high sensitivity to drugs targeting the VEGR pathway (16). Cabozantinib has a different drug target profile (VEGFR, MET, AXL) compared to the other inhibitors discussed in this review. This suggests that the alternate RTK targets may be (more) important in OS and ES, at least in some of those patients, although we do note that the PFS of cabozantinib did not differ much from the other drugs. Of the other drugs with largely overlapping abilities to inhibit VEGFR, PDGFR and FGFR signaling, regorafenib, at least as a single agent, seems to be an interesting option given its reported responses in both OS and ES patients. The differences in CBR, ORR and PFS between these very similar drugs are however small with overlapping confidence intervals between studies, and not all of these drugs have been trialed to the same extend or on the exact same patient population to allow a truly unbiased and direct drug comparison. Without an actual effect on survival, or further elucidation of predictive biomarkers, it remains challenging to state which drug would work best for which patient. From a practical point of view, not in the least based on its worldwide clinical application in other sarcomas, pazopanib is worthy of further consideration for OS and ES. There are at present however no active clinical trials that further test pazopanib in these sarcoma subtypes. The vast majority of currently active clinical trials in this field are centered around regorafenib and cabozantinib, either as a monotherapy, or in combination with chemotherapy or immunotherapy. The combination of anti-angiogenic multi-RTK inhibitors to immune checkpoint inhibitors is interesting for OS and ES, as the vast majority of these drugs are capable of modulating the tumor microenvironment in such a way that it boosts the efficacy of anti-PD-1 therapy (8). Of the anti-angiogenic multi-RTK combination therapies clinically trialed in OS so far, the efficacy and tolerability of lenvatinib combined with etoposide and ifosfamide is of interest. The currently ongoing trial comparing the efficacy and safety of ifosfamide and etoposide with or without lenvatinib in children and AYA OS patients is expected to deliver valuable insights into future directions of OS treatments. For ES patients, the combination of anlotinib with chemotherapy seems, based on the data available, also a promising avenue. Overall, the clinical results observed with anti-angiogenic multi-RTK inhibitors in OS and ES are interesting given the fact that these drugs were all tested on heavily pre-treated patients with a particularly poor prognosis. However, we do note that although clinical benefit has been reported, improvements in PFS were only modest, and so far no survival benefit was reported. Additionally, the number of patients entered in the studies was limited due to the rarity of the tumors and the challenges to perform studies with tyrosine kinase inhibitors in these rare sarcomas. Single agent anti-angiogenic multi-RTK inhibitors have side effects related to the target inhibition. Combination treatments with different chemotherapy regimens and multi-RTKs regimens in these heavily pretreated patients have shown to come with a price of increased toxicity necessitating dose reductions. Careful attention should be paid to which (standard-of-care) chemotherapies or other drugs these anti-angiogenic multi-RTK inhibitors are combined, and prospective randomized trials are important to determine both the exact added value of the combination strategy, as well as the associated toxicity. Another factor that deserves further consideration, is the timing of the introduction of novel therapeutic avenues. For either a combined or single-agent multi-RTK treatment strategy, inclusion of molecular predictive biomarkers is expected to refine the group of expected responders, and avoid unnecessary serious side effects. Refinements in drug dosing, for example with therapeutic drug monitoring and carefully chosen treatment combinations can further reduce side effects.

## Conclusion

Altogether, anti-angiogenic multi-RTK inhibitors, alone or in combination with other drugs, represent an interesting option for OS and ES patients. Definitely, more research is required at an international level before clinical implementation is on the horizon. More investigations into predictive response biomarkers, and the conduction of prospective randomized clinical trials, will be important to maximize treatment efficacy, reduce drug toxicity and compare the anti-angiogenic multi-RTK-targeted treatment regimen to a relevant comparator arm to adequately assess its potential benefit. Reimbursement of these drugs can only be facilitated after robust and ideally randomized clinical trials, including health-related quality of life assessments to incorporate the patient’s perspective in the best possible way.

## Author contributions

Study conceptualization and writing first draft performed by EF. EF, MV, and WG contributed substantially in literature search, interpretating the data, drafting the final version and revising the text and therefore agree to be accountable for all aspects of the work in ensuring that questions related to the accuracy or integrity of any part of the work are appropriately investigated and resolved. All authors contributed to the article and approved the submitted version.
